# Transcriptomic landscape of cumulus cells from patients <38 years old with a history of poor ovarian response (POR) treated with platelet-rich plasma (PRP)

**DOI:** 10.18632/aging.206202

**Published:** 2025-02-18

**Authors:** Leah M. Roberts, Nola Herlihy, Andres Reig, Shiny Titus, Rolando Garcia-Milian, James Knight, Raziye Melike Yildirim, Cheri K. Margolis, Yigit Cakiroglu, Bulent Tiras, Christine V. Whitehead, Marie D. Werner, Emre Seli

**Affiliations:** 1 IVIRMA Global Research Alliance, IVIRMA New Jersey, Basking Ridge, NJ 07920, USA; 2 Bioinformatics Support Hub, Cushing/Whitney Medical Library, Yale School of Medicine, New Haven, CT 06520, USA; 3 Yale Center for Genome Analysis, Yale University, New Haven, CT 06520, USA; 4 Department of Obstetrics and Gynecology, Acibadem Mehmet Ali Aydinlar University, Istanbul, Turkey; 5 Department of Obstetrics, Gynecology and Reproductive Sciences, Yale School of Medicine, New Haven, CT 06520, USA

**Keywords:** plasma rich protein, diminished ovarian reserve, RNA-sequencing

## Abstract

Intraovarian injection of autologous platelet-rich plasma (PRP) has recently been investigated as a potential treatment for patients with diminished ovarian reserve. In the current study, differential gene expression in cumulus cells obtained from patients treated with PRP was compared to controls. RNA sequencing libraries were constructed from the cumulus cells, and differential expression analysis was performed with a false discovery rate threshold of *p*-value ≤0.05 and Log2 fold change ≥0.584. RNA sequencing of cumulus cells revealed significant differences in gene expression when comparing those treated with PRP and resulted in a live birth (*n* = 5) to controls with live birth (*n* = 5), or to controls with failed implantation (*n* = 5). Similarly, when all samples treated with PRP (those that resulted in live birth or arrested embryos (*n* = 10)) were compared to all samples from controls (those that resulted in live birth, no pregnancy, or arrested embryos (*n* = 13)), gene expression was significantly different. Several pathways were consistently affected by PRP treatment through multiple comparisons, including carbohydrate metabolism, cell death and survival, cell growth and proliferation, and cell-to-cell signaling, all of which have been implicated in human causes of infertility.

## INTRODUCTION

Poor ovarian response (POR), also termed as diminished ovarian reserve (DOR), delineates a clinical scenario where decreased quantity of oocytes that remain in the ovary result in suboptimal response to ovarian stimulation in women undergoing infertility treatment with *in vitro* fertilization (IVF) [1]. While the perfect definition of POR/DOR remains to be agreed upon, these patients account for an increasingly large portion of IVF cycles and suffer from significantly worse outcomes [2]. Indeed, patients diagnosed with DOR account for approximately 1/4 of cycles in women undergoing IVF in the United States, and experience a lower (21% vs. 36%) cumulative live birth rate per retrieval cycle initiated compared to all other patients [3, 4]. A more restrictive approach to diagnose POR has been proposed as the ESHRE Bologna Criteria, which requires a patient to meet two out of three of the following conditions: age ≥40 years or any other risk factor for POR; an abnormal ovarian reserve test (AMH <0.5–1.1 ng/mL or AFC of 5–7); and a prior cycle with ≤3 oocytes retrieved [5]. Another commonly used definition for POR is based on POSEIDON criteria, which includes patient age, ovarian reserve parameters and prior response to stimulation [6]. In 2011, 15% of IVF cycles performed in the United States met Bologna Criteria for POR, and these cycles achieved a live birth rate per cycle start of 4% and a cumulative live birth rate of only 17% [3]. As there is a proven association between the number of oocytes retrieved and cumulative live birth rate, a number of experimental treatment strategies have been used to try to increase the oocyte yield in these patients to maximize their chances of success [7].

Platelet-rich plasma (PRP) is a concentrated form of plasma obtained by the centrifugation of whole blood to remove red blood cells, creating a concentrated sample of platelets [8, 9]. PRP is thought to enhance healing due to its increased concentration of growth factors, chemokines, and cytokines. The mechanisms by which these factors influence cellular behavior have not yet been fully characterized, however, proposed mechanisms include induction of neoangiogenesis, promotion of cellular migration, cell proliferation and tissue remodeling, and reduction of apoptosis [9, 10].

Injection of autologous PRP as a therapeutic intervention has been used in diverse fields including plastic surgery, dermatology, dentistry, wound healing, orthopedic surgery, and cardiothoracic surgery [8]. Cohort studies have also been performed within the reproductive medicine space using PRP for endometrial hypoproliferation [11, 12], recurrent implantation failure [11, 13], as well as poor ovarian response (POR) and primary ovarian insufficiency (POI) [9, 10, 14–19].

In most studies evaluating the impact of PRP on POR and POI, ovarian reserve parameters, serum follicle stimulating hormone (FSH), anti-mullerian hormone (AMH), and antral follicle count (AFC) were evaluated in the pre- and post-treatment cycles to determine the effects of PRP [8, 9, 14, 15, 18, 19]. These series have shown a variable decrease in FSH and an increase in AMH and AFC, some of which were statistically significant [10, 18, 19]. Similarly, ovarian injection of PRP in women with POR and POI was associated with an increased number of oocytes and embryos [10, 18, 19], and improved embryo euploidy rates were observed in a pilot study [20]. Most recently, two recent randomized clinical trials (RCTs) investigating the efficacy of autologous intraovarian PRP injection in patients with POR failed to demonstrate an increase in pregnancy or live birth rates, while they reported an improvement in ovarian reserve parameters [21], or the number of oocytes retrieved [22]. The latter trial was criticized in regards to storage of PRP prior to use, and intramedullary instead of (sub) cortical injection, as well as for being underpowered [23]. Besides, none of these studies investigated the ovarian molecular pathways that may be affected by PRP treatment.

This study aimed to determine how PRP affects follicular environment by identifying genes that are differentially expressed in cumulus cells of POR patients treated with PRP. Our findings indicate that PRP treatment regulates certain pathways that could contribute to follicular activation and oocyte maturation. Our ultimate aim is to identify specific factors that can be selectively used for follicular activation *in vivo* or *in vitro*.

## RESULTS

### Study population

Cumulus cell samples were classified based on the treatment received by the patient (PRP vs. no PRP) and the outcome of the embryo (live birth after single euploid embryo transfer, no pregnancy after single euploid embryo transfer, or embryonic arrest in culture at blastocyst stage).

Group one consisted of cumulus cell samples from oocytes that resulted in a euploid embryo and live birth upon transfer in patients who were in the control group, and therefore did not receive PRP treatment (C-LB; *n* = 5). Group two consisted of cumulus cell samples from oocytes that resulted in a euploid embryo and livebirth upon transfer in patients who received PRP treatment (PRP-LB, *n* = 5). Group three consisted of cumulus cell samples from oocytes that resulted in a euploid embryo, which failed implantation upon transfer in patients who did not receive PRP treatment (C-NP, *n* = 3). Group four consisted of cumulus cell samples from oocytes that resulted in embryos arrested after reaching the blastocyst stage in control patients who did not receive PRP (C-ARR, *n* = 5). Group five consisted of cumulus cell samples from oocytes that resulted in embryos arrested after reaching the blastocyst stage in patients treated with PRP (PRP-ARR, n-5). Embryos in group four and five were not suitable for trophoectoderm biopsy/PGT-A and cryopreservation for future use. There were 23 cumulus cell samples analyzed by RNA sequencing from 18 different patients.

The baseline at the time of randomization and IVF cycle characteristics of the patients associated with the evaluated cumulus cells are presented in Table 1. No statistically significant difference, using an unpaired parametric *t*-test with welch correction, was found in the mean age, AMH, FSH, AFC, BMI, total motile sperm count (TMSC) of partner, or euploid embryos created within the patients of the samples used for this analysis.

**Table 1:** Patient characteristics.

	**PRP (7)**	**No PRP (12)**	***P*-value**
Age (y) mean *Std dev* (95% CI)	32.2 *4.5* (28.0–36.3)	34.3 *1.9* (33.1–35.5)	0.27
BMI (kg/m^2^)	22.7 *3.4* (19.6–25.8)	23.9 *3.4* (20.9–27.0)	0.51
TMC (in Millions)	123.6 *162* (0–273)	89.3 *62.6* (49.5–129)	0.61
AMH (ng/mL)	1.327 *0.5* (0.8–1.8)	0.9 *0.6* (0.6–1.3)	0.13
FSH (mIU/mL)	8.5 *2.3* (6.4–10.7)	8.5 *2.7* (6.7–10.2)	0.96
AFC	9.1 *4.3* (5.2–13.1)	7.5 *3.0* (5.6–9.4)	0.40
Total FSH medication used	2458 *908* (1505–3411)	3458 *1175* (2617–4298)	0.08
Max E2 (pg/mL)	2973 *2620* (550–5396)	1832 *740* (1362–2302)	0.30
MII	5.7 *3.7* (2.3–9.2)	4.8 *2.3* (3.4–6.3)	0.58
2PN	4.6 *3.3* (1.6–7.6)	3.8 *2.0* (2.5–5.1)	0.60
Embryos	3 *2.8* (0.4–5.6)	1.8 *1.2* (1.0–2.5)	0.30
Euploid embryos created	2.6 *2.8* (0–5.2)	1.6 *1.1* (0.9–2.3)	0.40

There were no *P* < 0.05. Abbreviations: BMI: body mass index; TMC: total motile count; AMH: anti-mullerian hormone; FSH: follicle-stimulating hormone; AFC: antral follicle count; Max E2: maximum estrogen; MII: metaphase two/mature oocyte; 2PN: two pronuclei/normally fertilized oocyte.

### Comparison of PRP-treated cumulus cells that resulted in live birth to untreated controls that resulted in livebirth

We first compared cumulus cell samples from patients treated with PRP, which led to a euploid embryo transfer and live birth (Group 2), to those who achieved the same outcome without PRP treatment (Group 1). This comparison allowed us to study the molecular impact of PRP treatment independent of other factors associated with viability.

**Figure 1:**
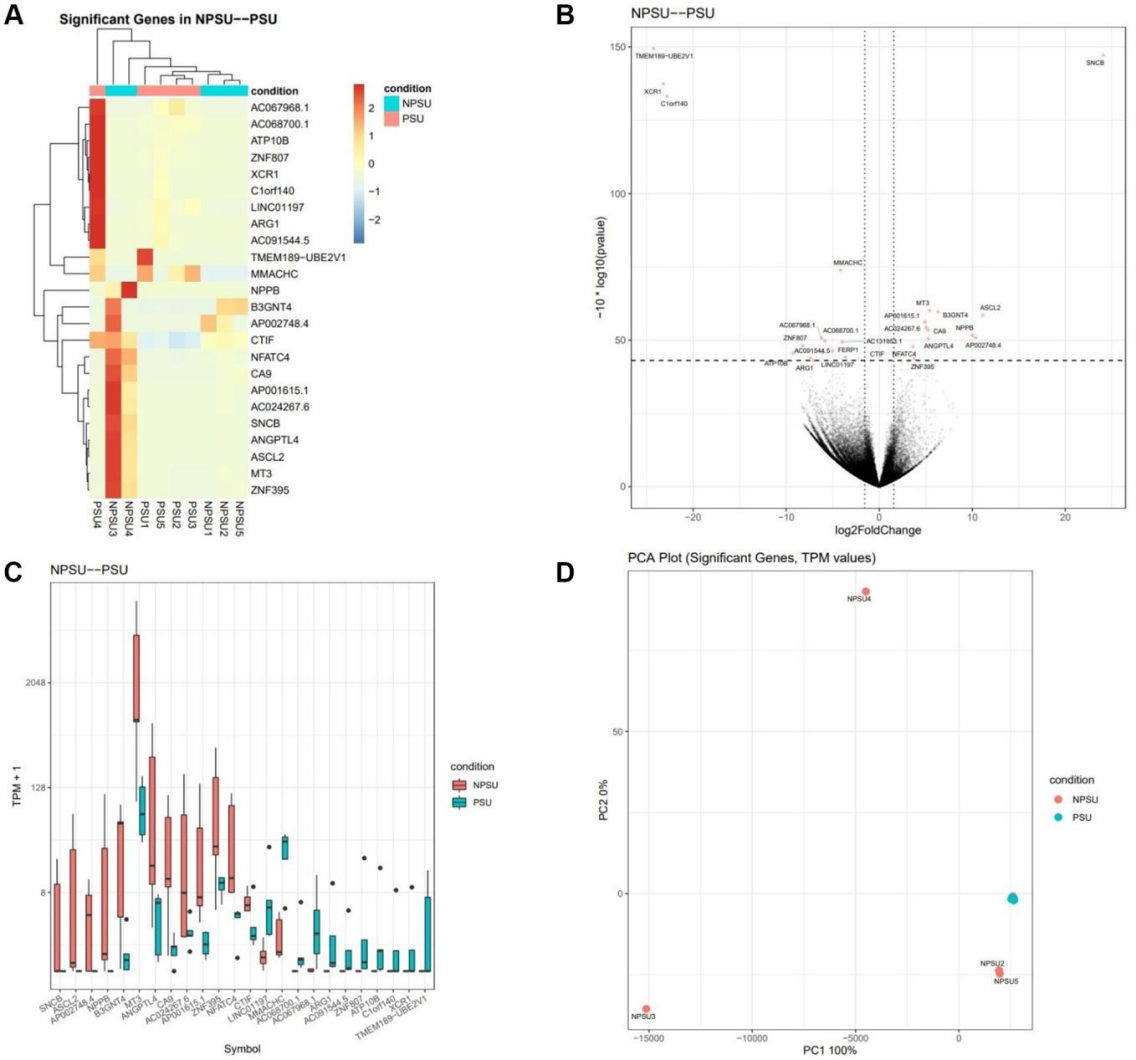
**Gene expression is altered in PRP-treated patients with sustained implantation (Group 2) compared to control patients with sustained implantation (Group 1).** (**A**) The heat map illustration shows differentially expressed genes. The color spectrum ranging from red to blue indicates normalized levels of gene expression from high to low. (**B**) Volcano plot for RNA-seq comparing Group 2 to Group 1. (**C**) Differentially expressed genes in Group 2 compared to Group 1, *P* < 0.05 for each. For the box plots, the bottom and top whiskers denote 5 and 95 percentile values, the bottom and top bounds of the rectangle denote the 25 and 75 percentile values, and the line in between denotes the median (50 percentile) value of the distribution. (**D**) PCA plot for RNA-seq for significant genes comparing Group 2 to Group 1. The transcripts per million (TPM) value represents the relative expression level comparable between samples.

**Figure 2:**
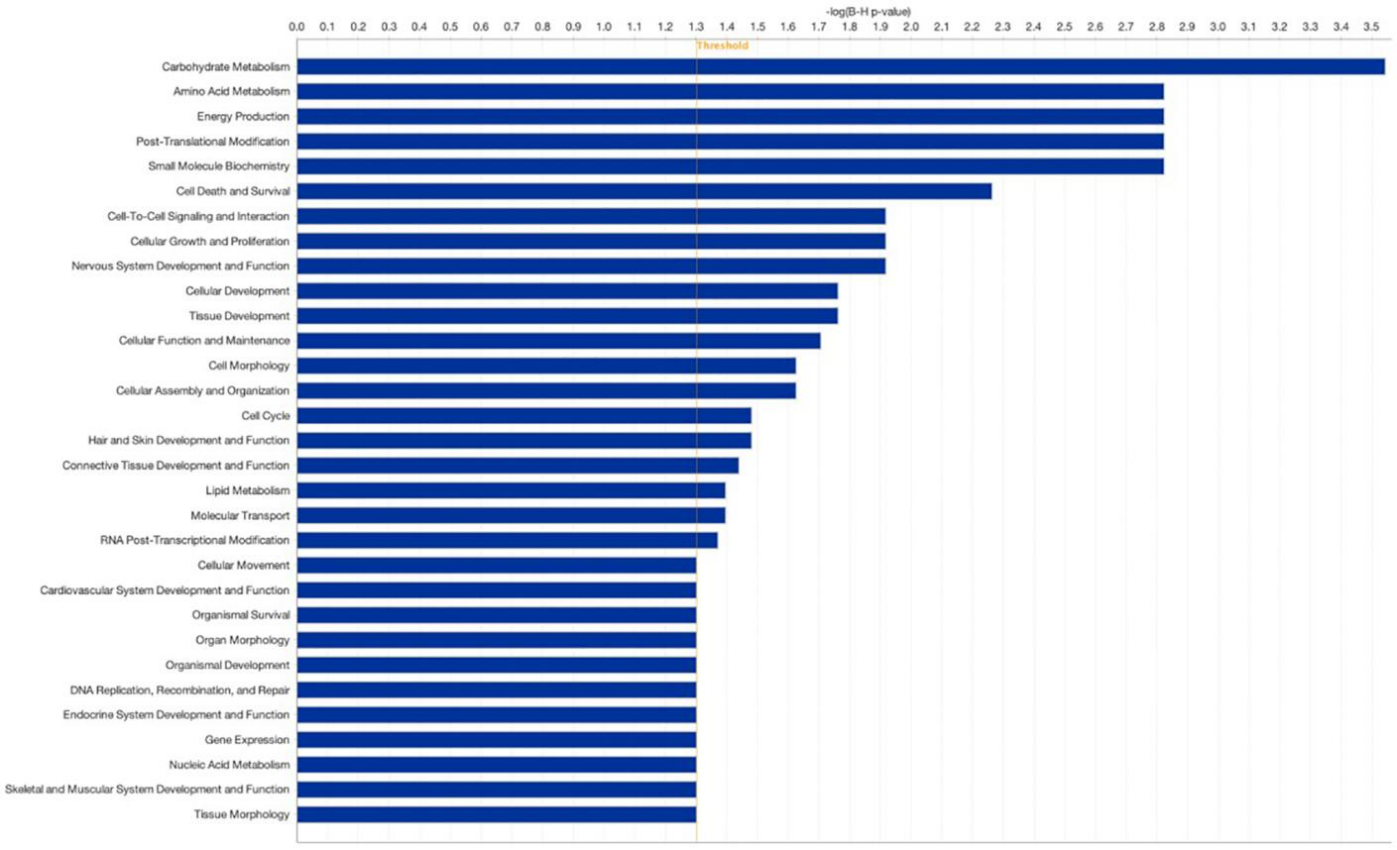
**Pathway analysis comparing patients with sustained implantation (Group 2) to control patients with sustained implantation (Group 1).** Pathway analysis was performed using the Gene Ontology bioinformatics tool. Log2 fold change (FC) ≥0.584 false discovery rate (FDR).

Hierarchical clustering of the DEGs partitioned into two distinct clusters showed differential gene expression between the two groups suggesting high reproducibility of the sequencing data. The comparison of Group 2 vs. Group 1 revealed a total of 24 significantly DEGs (FDR 0.05); 13 were upregulated, and 11 were downregulated. For genes that were over-expressed, the FC ranged from 1.6 to 24. For under-expressed genes, the FC ranged from −3.6 to −24.2. Genes affected included those involved in carbohydrate metabolism, amino acid metabolism, energy production, post-translational modification, cell death and survival, cell-to-cell signaling and interaction, and cellular growth and proliferation (Figures 1, 2).

### Comparison of all cumulus cells samples from patients treated with PRP to those that were not treated with PRP

**Figure 3:**
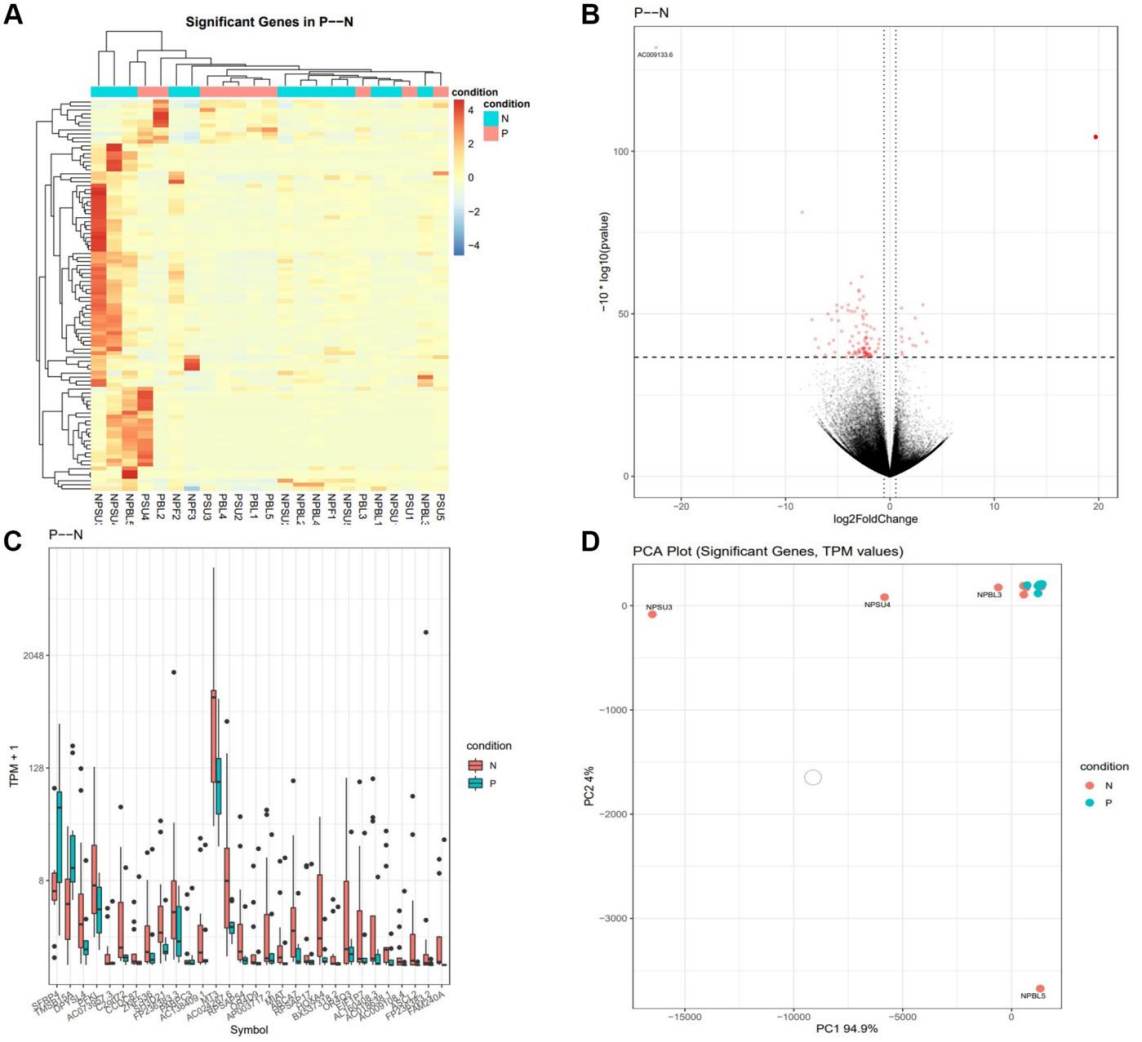
**Gene expression is altered in cumulus cells of PRP-treated patients (Groups 2 and 5 combined) compared to controls (Groups 1, 3, and 4 combined).** (**A**) The heat map illustration shows differentially expressed genes. The color spectrum ranging from red to blue indicates normalized levels of gene expression from high to low. (**B**) Volcano plots for RNA-seq comparing PRP to control. (**C**) Differentially expressed genes in CONT and PRP, *P* < 0.05 for each. For the box plots, the bottom and top whiskers denote 5 and 95 percentile values, the bottom and top bounds of the rectangle denote the 25 and 75 percentile values, and the line in between denotes the median (50 percentile) value of the distribution. (**D**) PCA plots for RNA-seq for significant genes comparing PRP to control. The transcripts per million (TPM) value represents the relative expression level comparable between samples.

**Figure 4:**
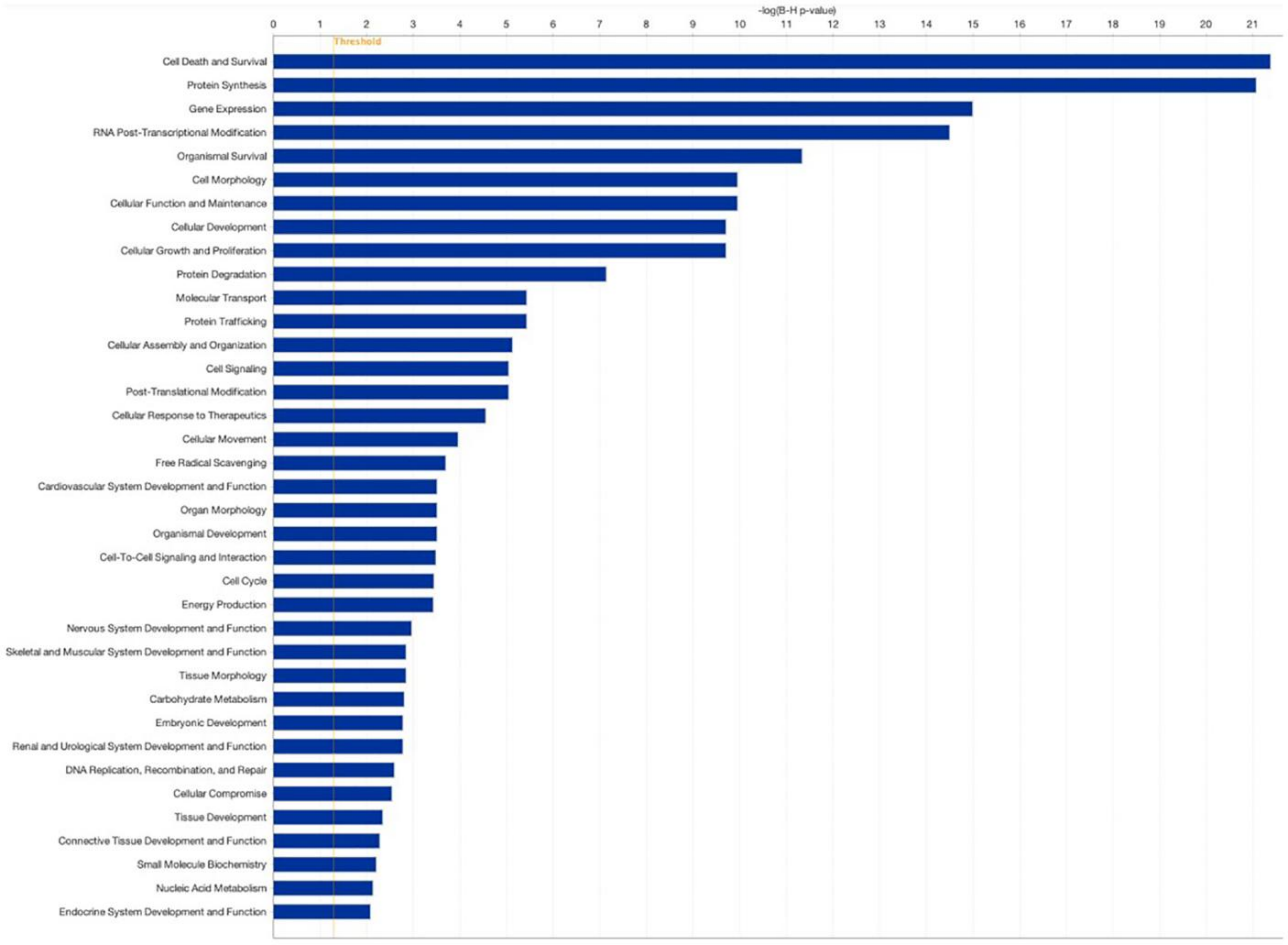
**Pathway analysis comparing patients treated with PRP (Groups 2 and 5 combined) to controls (Groups 1, 3, and 4 combined).** Pathway analysis was performed using the Gene Ontology bioinformatics tool. Log2 fold change (FC) ≥0.584 false discovery rate (FDR).

To further assess the impact of PRP on follicular somatic cell gene expression, and to test whether similar gene pathways would be affected in this expanded analysis, we compared all samples from patients treated with PRP (Groups 2 (PRP-LB) and 5 (PRP-ARR)) to all samples from the patients in the control group that did not receive PRP injection (Groups 1 (C-LB), 3 (C-NP), and 4 (C-ARR)). The comparison revealed a total of 98 significant DEGs; 12 were upregulated, and 86 were downregulated. For genes that were over-expressed, the FC ranged from 1.1 to 3.2. For under-expressed genes, the FC ranged from −0.6 to −8.4. Genes affected include those involved in cell death and survival, protein synthesis, gene expression, pre-and post-transcriptional modification, organismal survival, cell morphology, cellular function and maintenance, cellular development, cellular growth and proliferation, and embryonic development (Figures 3, 4).

### Comparison of PRP treated cumulus cells that resulted in live birth to untreated controls that did not result in a pregnancy

**Figure 5:**
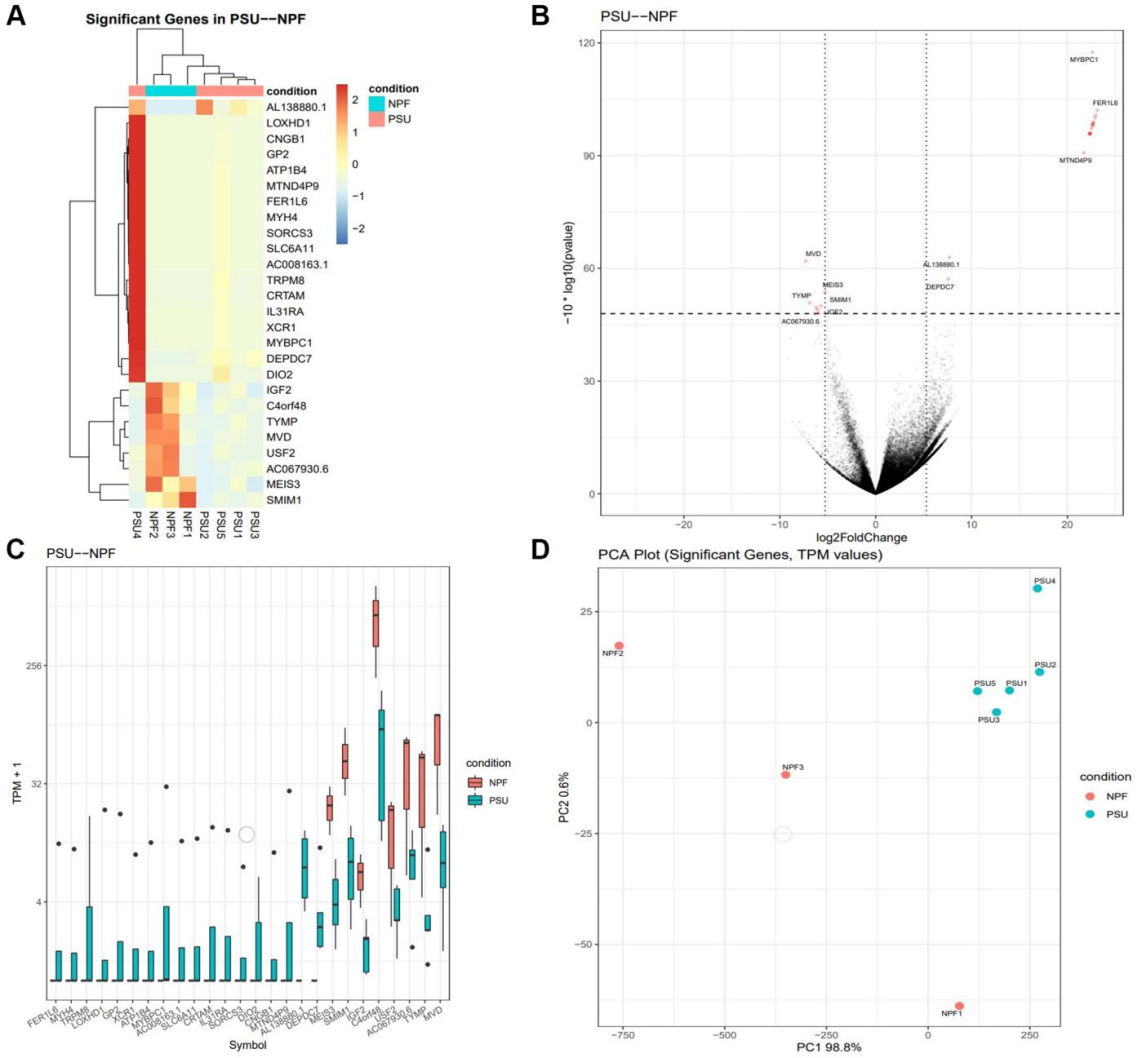
**Gene expression is altered in PRP-treated patients with sustained implantation (Group 2) compared to control patients without sustained implantation (Group 3).** (**A**) The heat map illustration shows differentially expressed genes. The color spectrum ranging from red to blue indicates normalized levels of gene expression from high to low. (**B**) Volcano plots for RNA-seq comparing PRP-LB with C-NP. (**C**) Differentially expressed genes in Group 2 versus Group 3, *P* < 0.05 for each. For the box plots, the bottom and top whiskers denote 5 and 95 percentile values, the bottom and top bounds of the rectangle denote the 25 and 75 percentile values, and the line in between denotes the median (50 percentile) value of the distribution. (**D**) PCA plots for RNA-seq for significant genes comparing Group 2 with Group 3. The transcripts per million (TPM) value represents the relative expression level comparable between samples.

**Figure 6:**
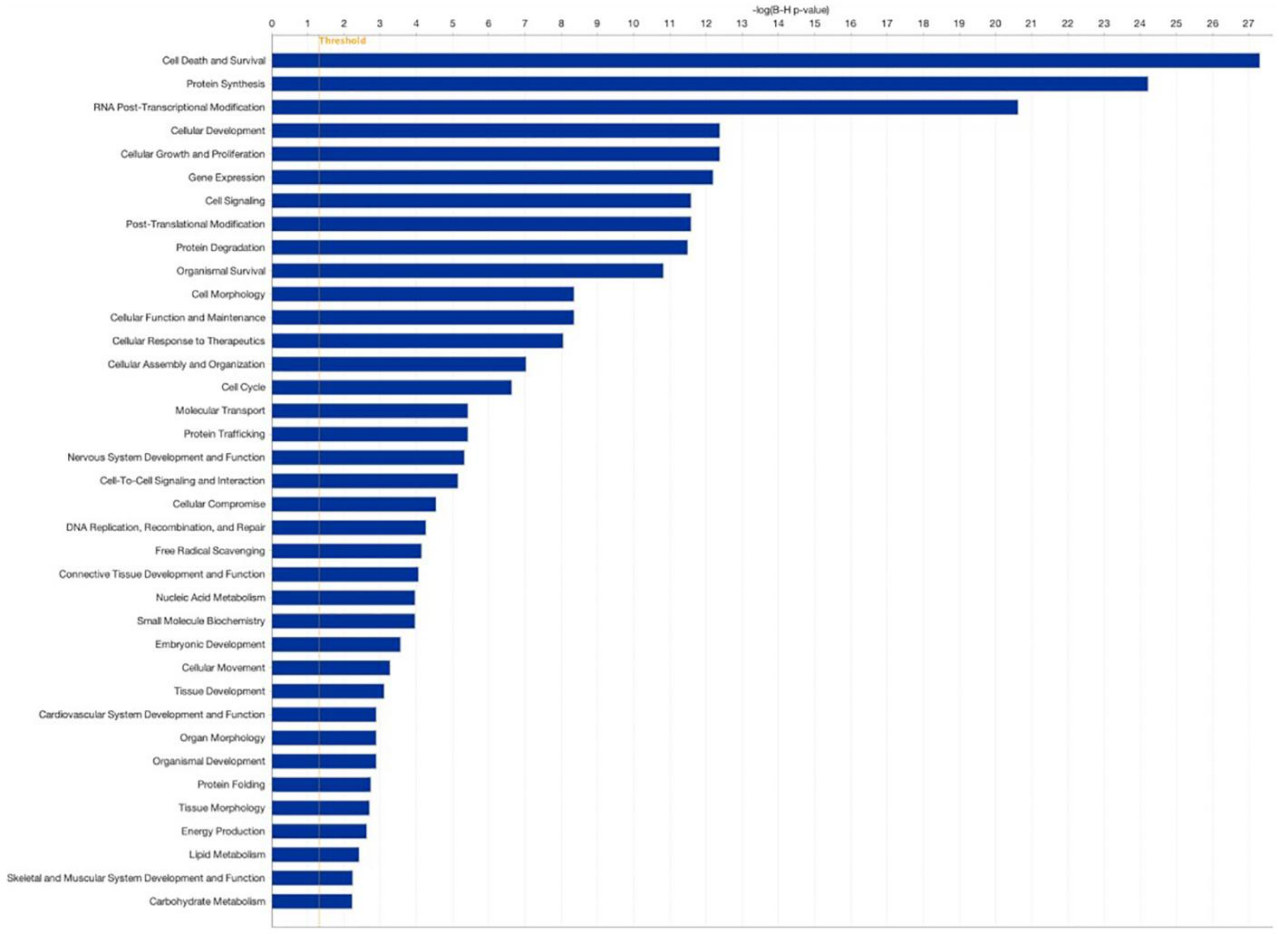
**Pathway analysis comparing patients with sustained implantation (Group 2) to control patients without sustained implantation (Group 3).** Pathway analysis was performed using the Gene Ontology bioinformatics tool. Log2 fold change (FC) ≥0.584 false discovery rate (FDR).

Finally, a comparison was made between the extremes of PRP treatment followed by livebirth (Group 2 (PRP-LB)) vs. no PRP and failed implantation (Group 3 (C-NP)) revealing a total of 26 significant DEGs. 18 were upregulated, and 8 were downregulated. For genes that were over-expressed, the FC ranged from 7.6 to 23.1. For under-expressed genes, the FC ranged from −5.3 to −7.3. Genes affected included those involved in cell death and survival, protein synthesis, cellular development, cellular growth and proliferation, cell-to-cell signaling, and embryonic development (Figures 5, 6).

### RNA sequencing defined repertoire of differentially expressed genes

Quantitative RT-PCR demonstrated agreement with the RNA-seq relative expression data (Supplementary Figure 1). Reference gene *GAPDH* was used as the internal control. Of the genes shown to be differentially expressed in the RNASeq analysis, the genes MMACHC and ANGPTL4 were chosen for validation with RT-PCR. These both showed appropriate changes with MMACHC showing upregulation (increase) in Group 2 samples (*p*-value 0.0286) and ANGPLT4 showing downregulation (decrease) in the Group 2 samples (*p*-value 0.0317) which was consistent with what was seen in the RNASeq analysis and successfully validated our results (see Supplementary Figure 1).

## DISCUSSION

In comparing patients treated with PRP to control patients, altered expression of several genes was detected and several pathways associated with embryo development emerged as potential targets of PRP treatment. In our three comparisons, there was significant consistency in the pathways identified as being affected by the PRP treatment. These include pathways involved in carbohydrate metabolism, cell death and survival, cell growth and proliferation, and cell-to-cell signaling. Comparison of Group 2 to Group 1 was especially important, as it allowed us to identify the genes that are affected by this treatment, and not those differentially regulated as a factor of viability. The comparison of all PRP patients to all control patients was important to confirm that these same pathways were conserved in being affected by the treatment. The comparison of Group 2 to Group 3 provided mechanistic insight into how this treatment can affect embryo development. Together, these three comparisons provide a more complete picture of how PRP’s effects on ovarian biology are mediated.

This study identified carbohydrate metabolism as the most affected pathway when comparing Group 2 to Group 1. In addition, the other two comparisons also detected this pathway as being differentially regulated after PRP treatment. These findings could have two important implications. First, our findings support PRP’s effect on carbohydrate metabolism across three experimental designs involving different sample sources. Second, they provide a possible mechanistic insight into how PRP may help regulate follicle development and oocyte maturation. Cumulus cells support the oocyte in a variety of ways and are vital in regulating substrate utilization and transport [24, 25]. In terms of carbohydrate metabolism within the cumulus oophorus complex (COC), the oocyte relies on pyruvate as it cannot metabolize glucose. Glycolysis occurs after circulating glucose enters the cumulus cells and the pyruvate generated in cumulus cells is then passed on to the oocyte through gap junctions or active transport (reviewed in [25]). Pyruvate is used as a primary energy source for the oocyte and the early preimplantation embryo (cleavage stage), and the extent of pyruvate uptake has been proposed as a biomarker predictive of embryo viability [25–28]. A study by Xie showed that when genes involved in glucose metabolism like Glucose-6-phosphate dehydrogenase (*G6pd*) and mitochondrial pyruvate carrier 1 (*Mpc1*) are silenced in mouse cumulus cells using RNAi, the maturation ability of their co-cultured oocyte is significantly impaired [29]. It is also known that patients with pathogenic variants in *PMM2* genes or genes affecting the Leloir pathway (such as in *GALT, GALM*, or *GALE*), often have POI [30–32]. In showing that carbohydrate metabolism pathways are significantly changed by PRP treatment, we have insight into a potential mechanism for how PRP could be supporting follicular development and for other PRP treatments in general that can be further explored and targeted to develop more specific treatment modalities.

Also affected were pathways in cell death and survival and cell growth and proliferation. Cell death and survival was the most affected pathway when comparing all PRP to control as well as Group 2 compared to Group 3 and was also identified as highly affected in Group 2 compared to Group 1. Cellular growth and proliferation were also highly affected in all three comparisons. These pathways have been implicated in infertility and are affected in multiple knockout animal models of infertility and subfertility [33–36]. For instance, *Clpp* mutations have been seen in Perrault syndrome which causes sensorineural hearing loss and early ovarian failure in humans [30–37]. Analysis of gene pathways affected in the absence of *Clpp* shows a significant over-representation of pathways involved in the regulation of cell death and growth [33]. *Clpp* knockout mice create lower numbers of mature oocytes, fail to generate blastocysts, and have accelerated depletion of follicular reserve [33]. PRP’s influence on these pathways could conceivably be the cause of its effects on the ovaries.

Also widely affected in all three comparisons were pathways in cell-to-cell signaling. One important signaling molecule between oocytes and their surrounding supportive cells is BMP15 [38]. BMP15 genetic alterations have been noted in cases of POI and infertility in humans [39]. It is a cause of infertility in a variety of different species from natural sheep to knockout porcine models, with drastically decreased volume and degradation of follicles noted [40]. Analysis of genes and pathways affected by the absence of mitochondrial fusion 1 (MFN1) has shown significant over representation in the regulation of adherence junction signaling and death receptor signaling [35]. *Mfn1* knockout mice have significantly smaller ovaries (1.65 um2 versus 4.8 um2 *p* < 0.05), and fail to produce mature oocytes or have any pregnancies or deliveries [35]. Embryonic Poly(A)-Binding Protein (EPAB) is another protein that has been established with knockout models to have both effects on cell signaling and folliculogenesis [36, 41, 42]. It is logical to infer that PRP may affect ovarian reserve by causing changes in the cell-to-cell signaling pathway within the follicle.

The variety and consistency of the pathways affected in this analysis are particularly intriguing in the context of treatment for POR. These pathways have been associated with folliculogenesis, ovarian reserve, ovarian volume, and infertility in both knockout mice and human populations [30, 37–39]. POR and POI are caused by the delicate interplay between the number of primordial follicles created in the developing embryo, the quality of these oocytes, and the rate of their depletion. While we cannot increase the ovarian reserve in a patient already diagnosed with POI or POR, we can potentially help these patients make the most of their remaining reserve and enhance oocyte yield during treatment by using therapies that target infertility-related pathways.

In patients who underwent single euploid embryo transfer with or without prior PRP treatment, and failed to achieve a pregnancy, the reason for implantation failure remains to be determined. The role of uterine factors in this process is highly debated as suggested in a number of recent publications [43, 44].

There are some strengths and limitations of the present study. Firstly, cumulus cells from each oocyte were individually collected- not pooled- and cryopreserved until analysis and they were independently sent for RNAseq. We believe this constitutes a significant strength of our manuscript. Secondly, our study focused on cumulus cells (not mural cells or both) because homogeneity of cumulus cells can more reliably be achieved, whereas collection of mural granulosa cells is often complicated with contamination with white blood cells that may have a completely different gene expression profile. As a limitation, in the RCT that the samples were derived from, patients with 3 or less oocytes retrieved (or had cycle cancelled due to poor response) in at least two prior IVF cycles were included as having POR. These individuals would be classified as POR based on POSEIDON sub-classifications. Subsequently, some of these patients had more than 3 oocytes retrieved in the cycle that they underwent as part of the study. This variation is a common occurrence in patients diagnosed with POR using POSEIDON or Bologna criteria, as shown in a recent large retrospective study and for this reason can be accounted as a limitation of the diagnosis of POR in our study [45]. Type of trigger (GnRH, hCG, or dual trigger) might also be another limitation of the study. In the RCT that the samples were derived from [21], GnRH, hCG, or dual trigger was administered in order to trigger follicle maturation. Although all trigger strategies work though upregulation of Epidermal growth factor (EGF)-like growth factors to promote the release of the cumulus oophorus complex, they could differentially affect gene expression in cumulus cells [46, 47]. However, even if cumulus cell gene expression is differentially affected by the type of trigger, it would increase variability and decrease the number of genes and pathways identified. Therefore, the pathways in the current study are likely to be independent of the type of trigger, and arguably more relevant. In summary, the injection of PRP into the ovarian cortex of patients with POI caused changes in several gene expression pathways in the cumulus cells. Future directions for research include evaluating and further exploring each of these pathways to delineate how PRP is affecting the cumulus cells. The ultimate aim would be to target specific components to create more precise treatments that can help improve fertility outcomes.

## MATERIALS AND METHODS

### Sample collection

Cumulus cell samples from PRP-treated and non-PRP-treated patients were obtained from the RMANJ (Reproductive Medicine Associates of New Jersey Basking Ridge, NJ, USA) biobank. These samples had been collected as part of a prior randomized controlled study investigating the impact of intraovarian autologous PRP injection on patients with POR undergoing IVF. All study procedures were approved by and conducted according to the Institutional Review Board (Advarra 2019). Before collecting samples for the initial study, all patients were informed, and written consent was obtained, which included sample preservation and usage in future research.

Adult patients between ages 18 and 37 who were diagnosed as POR were included in this study. POR was defined as having at least two IVF cycles that were either cancelled due to poor follicle development (<3) or resulted in the retrieval of three or fewer mature oocytes at maximum gonadotropin doses (450 FSH daily). Exclusion criteria included those who had a known genetic cause for POR, were planning to do preimplantation genetic testing for monogenic disorders (PGT-M) or structural rearrangements (PGT-SR), had increased risk of thrombosis, had ongoing malignancy, ongoing ovarian pathology (such as a dermoid cyst or endometrioma), had a history of gonadotoxic treatment or ovarian surgery, had a BMI >35, or had a diagnosis of endometrial insufficiency (<6 mm). Sperm sources (male partner or donor sperm) had to have at least 100,000 total motile sperm from an ejaculated sample.

Patients were randomized to either undergo an injection of 8 cc autologous PRP (4 cc per ovary) into their subcortical region in the cycle before their retrieval cycle or have no procedure (as a sham procedure was considered unnecessary risk). The technique was developed, optimized and described in prior cohort studies [18, 19], Briefly, patients who underwent the ovarian PRP procedure were placed under deep sedation, and then under transvaginal ultrasound guidance using a 35 cm 17G single lumen needle, 1 ml of PRP was injected into at least four different locations depending on the size of the ovaries underneath the ovarian cortex into each ovary. Injection was into the subcortical area where the most dormant follicles would exist. A 17 G needle was used in order to stabilize the ovaries and create a large enough space in the ovary with each puncture to allow delivery of PRP.

The cycle after randomization and either PRP procedure or no procedure, routine ovarian stimulation protocols with either microdose leuprolide flare cycle or gonadotropin-releasing hormone antagonist (ganirelix acetate 250 µg or cetrorelix acetate 0.25 mg) were used as determined by the participant’s primary physician. Transvaginal ultrasound and hormone monitoring were performed every few days until at least two follicles measuring 17 mm or wider were noted, at which point either a GnRH, hCG, or dual trigger was administered. Patients then proceeded to egg retrieval 36 hours later. Cycles were cancelled if there was no follicular development after twelve nights of gonadotropin administration at maximum dosage. Collection of otherwise previously discarded specimens was performed to allow for further analysis, as was done in this study.

Cumulus cells were isolated from each individual oocyte retrieved, after which the oocytes and embryos were individually cultured, to ensure an accurate correlation with embryo development and associated outcomes. Cumulus cells were aspirated from each well, washed in phosphate-buffered saline, centrifuged at 90s for 15,000 g, and then stored as a pellet at –80°C.

Cumulus cells were analyzed in 5 groups to delineate changes in mRNA expression in response to PRP treatment and subsequent embryo and pregnancy outcome:

Group 1: IVF without PRP treatment (control), euploid embryo transfer and livebirth (C-LB).
Group 2: IVF following PRP treatment, euploid embryo transfer and livebirth (PRP-LB).
Group 3: IVF without PRP treatment (control), euploid embryo transfer, no pregnancy (C-NP).
Group 4: IVF without PRP treatment (control), embryo arrested at the blastocyst stage (C-ARR).
Group 5: IVF following PRP treatment, embryo arrested at the blastocyst stage (PRP-ARR).

### Cumulus cell RNA extraction and library construction

The cumulus cells corresponding to each oocyte/embryo were thawed and assessed individually, with approximately 100–200 cells per sample in initial analyses. SMART-Seq v4 Ultra Low Input RNA Kit for Sequencing (Takara Bio, San Jose, CA, USA) was used to prepare RNA according to the user manual. RNA integrity of samples was tested on Agilent Bioanalyzer (Agilent Technologies, Santa Clara, CA, USA) before sequencing. Samples meeting the stringent criteria for RNA quality, with the RIN number (8 and above), and concentration, were processed for sequencing. mRNA was converted into cDNA using long-distance PCR for amplification (17 cycles for 100 cells). cDNA was purified using the Agencourt AMPure XP Kit (Beckman Coulter, Brea, CA, USA) Agilent High Sensitivity DNA Kit was used for quantification on an Agilent 2100 Bioanalyzer (Agilent Technologies, Santa Clara, CA, USA). Nextera XT DNA Library Preparation Kit (Illumina Inc., San Diego, CA, USA) was used for library preparation. RNA concentrations of samples derived from individual oocytes’ cumulus cells ranged from 6.94–85.6 ng/ul after library preparation and prior to indexing by qubit. This information has been added to the revised version of the manuscript.

### Sequencing

Samples were sequenced to a depth of 44 million read pairs, 100 nucleotide length reads per sample using an Illumina Rapid v2kit (75 cycles) on a NovaSeq 6000 Sequencing System (Illumina Inc., San Diego, CA, USA). Data was converted to FASTQ files using the bcl2fastq2 v1.8.4 software (Illumina Inc, San Diego, CA, USA)*.* The number of raw reads ranged from 22 M to 214 M reads per sample, with an average of 160 M reads per sample. One sample had 22 M reads, and one had 79 M reads, with the rest having >110 M reads per sample.

### Data analysis

The reads were trimmed for quality and aligned with the reference human genome hg19 with GENCODE annotation (GENCODE reference annotation for the human and mouse genomes; Nucleic Acid Research, October 24, 2018). STAR –2.7.8a was used for alignment, annotated with Ensembl Transcripts release 100 and StringTie and BallGown for transcript abundance estimation (transcript level expression analysis of RNA-sequencing experiments with HISAT, String Tie, and Ball gown [48]. DESEq2 was used for differential analysis, which, for most data sets, gives the highest estimate of power [49]. The genes were identified as differentially expressed if Log2 fold change (FC) ≥0.584 and adjusted Benjamini–Hochberg (B–H) false discovery rate (FDR) *P* ≤ 0.05 [50]. The statistical program R was used for downstream processing and visualization of data.

### Ingenuity pathway analysis

Ingenuity Pathway Analysis (IPA) Ingenuity Systems (QIAGEN, content version: 51963813, 2020, Redwood City, CA, USA) was used to carry out pathway analysis for differentially expressed genes (DEG) across samples. Each gene was mapped to its corresponding gene object in the Ingenuity Pathways Knowledge Base. The DEGs used in pathway analysis were determined between experimental and control groups by using a filtering criterion of Log2FC 0.584 or above and FDR P 0.05 or lower. IPA Core Analysis was used to generate a network showing the overlap between functions and differentially expressed genes (Log2Fc ≥0.584 FDR *P* ≤ 0.05) resulting from the comparison between the different groups, in which FDR (or adjusted *P*-value) refers to the *P*-value (calculated using the right-tailed Fisher’s Exact Test) that is used in the overrepresentation analysis. This analysis calculates the overlap (*P* ≤ 0.05) between the list of DEG and pathways to determine if subsets of genes associated with specific pathways are over-represented (or enriched) among DEG. The fold change refers to the cut-off used to identify a gene as differentially expressed, e.g. fold change modular value of 2 or above, and to be included in the pathway analysis. In addition, IPA calculates the z-score to infer the activation states (increased or decreased) of implicated pathways and biological functions. This inference is based on the experimentally observed causal relationships found in the biomedical literature between genes and those functions [51].

### Validation of RNAseq results by quantitative RT-PCR

A quantitative reverse transcription-polymerase chain reaction (qRT-PCR) was carried out to confirm the differential gene expression of genes identified as differentially expressed in the RNA sequencing analysis. cDNA was prepared using the established protocol of SMART-Seq v4 Ultra Low Input RNA Kit for Sequencing (Takara Bio, San Jose, CA, USA). Qubit (Invitrogen, Carlsbad, CA, USA), was used to measure cDNA concentration. A total of 5 ng of cDNA and 1 ng forward and reverse primers (see Supplementary Table 1 for primer sequences) was used per reaction with RT-PCR using PowerUp SYBR Green Master Mix on the ViiA7 real-time PCR machine (Applied Biosystems, Waltham, MA, USA) for gene expression analysis. The components were mixed thoroughly and briefly centrifuged. Then PCR cycling conditions were 95°C for 5 minutes followed by a 45-cycle run with an annealing temperature of 60°C. The reference gene GAPDH was used for normalization. At least two replicates were done of each reaction. ΔΔCt method was used for the calculation of the difference in the expression of genes.

### Statistical analysis

Study population demographics of the samples utilized in this analysis were analyzed using an unpaired parametric *t*-test with welch correction using the GraphPad Prism version 10 (GraphPad, San Diego, CA, USA). Normality of distribution was assessed using Kolmogorov-Smirnoff test. Assessed parameters were normally distributed. Differences between groups were considered significant when the *P*-value was < 0.05.

### Data availability statement

The data underlying this article will be available in the article. Raw sequencing data can be made available by contacting the corresponding author.

## Supplementary Materials

Supplementary Figure 1

Supplementary Table 1
