# Supplementary material for: Transcriptomic landscape of cumulus cells from patients <38 years old with a history of poor ovarian response (POR) treated with platelet-rich plasma (PRP)
Source: Aging (Albany NY). 2025 Feb 18;17(2):431–47. doi: 10.18632/aging.206202 (PMC11892918; doi:10.18632/aging.206202)
Supplement: Supplementary Figure 1 [file aging-17-206202-s001.pdf]

SUPPLEMENTARY FIGURE

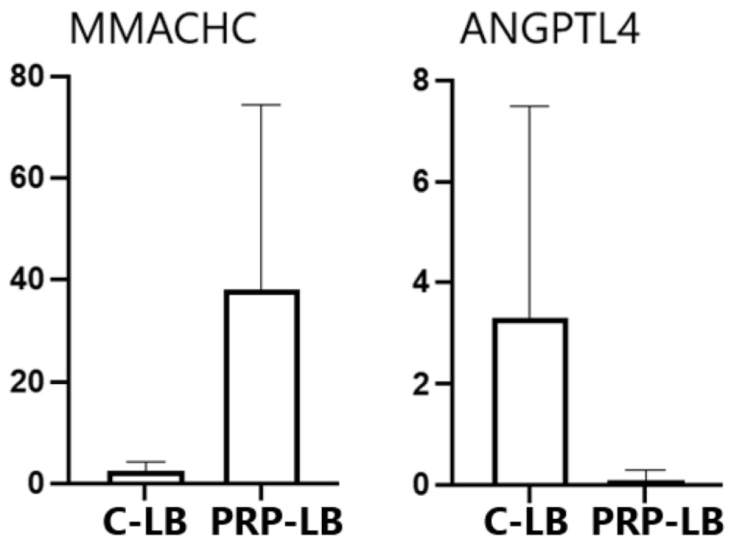

Supplementary Figure 1. RT-PCR results of the two confirmatory genes (MMACHC and ANGPTL4) for validation of RNA-Seq results. MMACHC Mann-Whitney test *p*-value 0.0286, ANGPTL4 *p*-value 0.0317.
