# Supplementary material for: Transcriptomic landscape of cumulus cells from patients <38 years old with a history of poor ovarian response (POR) treated with platelet-rich plasma (PRP)
Source: Aging (Albany NY). 2025 Feb 18;17(2):431–47. doi: 10.18632/aging.206202 (PMC11892918; doi:10.18632/aging.206202)
Supplement: Supplementary Table 1 [file aging-17-206202-s002.pdf]

SUPPLEMENTARY TABLE

Supplementary Table 1. The primers used for RT-PCR analysis for confirmation of validity of RNA-Seq results.

**ANGPTL4**	
Forward primer	GTCCACCGACCTCCCGTTA
Reverse primer	CCTCATGGTCTAGGTGCTTGT
**MMACHC**	
Forward primer	ATGGAGCCGAAAGTCGCAG
Reverse primer	CTGGAAGGGGTAAACCTCGAA
